# High Cognitive Load Situations With Different Conversation Topics Affect Walking Speed and Cognitive Complexity

**DOI:** 10.1093/geroni/igab046.2625

**Published:** 2021-12-17

**Authors:** Hyeon Jung Kim, Jennifer Yentes, Dawn Venema, Julie Blaskewicz Boron

**Affiliations:** 1 University of Nebraska Omaha, Omaha, Nebraska, United States; 2 University of Nebraska at Omaha, University of Nebraska at Omaha, Nebraska, United States; 3 University of Nebraska Medical Center, Omaha, Nebraska, United States

## Abstract

Walking and talking on the phone are common high-cognitive-load-situations (HCLS; e.g. dual-tasks), requiring extra attentional allocation and increasing perceived stress. We explored whether two load types, 1) single-task (ST) walking or talking on a phone and 2) HCLS walking while talking on a phone, influenced walking and/or cognitive performance among young (n=7; age=23.00±2.08yrs), middle-aged (n=14; age=44.79±7.42yrs), and older (n=15; age=74.47±3.91yrs) adults while controlling for perceived stress. Participants completed 3-minute trials of single-task walking (ST-W), single-task phone conversations with common (e.g., weather; ST-C) and uncommon topics (e.g., life experience; ST-U), and walking while talking on a phone (HCLS-C and HCLS-U). Walking speed was analyzed with 3(ST-W;HCLS-C;HCLS-U) x 3(Age) ANCOVA. HCLS resulted in slower walking speed (p<.001). Older adults exhibited slower speed across conditions compared to young (p=.015). Cognitive complexity (i.e., conversational tone and words greater than six letters (SIXLTR)) on the Linguistic Inquiry and Word Count (LIWC) were analyzed with 2(Cvs.U) x 2(STvs.HCLS) x 3(Age) ANCOVAs. Older age was associated with less cognitive complexity; positive tone (p=.014) and SIXLTR (p=.016), respectively in conversations. Uncommon topics reduced positive tone (p=.022) and SIXLTR (p=.003). Effects of HCLS on tone (p=.040) and SIXLTR (p=.005) varied with age. HCLS with different conversation topics resulted in reduced walking and cognitive complexity while controlling for perceived stress. The analysis of cognitive complexity using common/uncommon conversation topics is a novel method to assess the impact of HCLS. This research will disrupt the transformation of aging leading to a better understanding of attentional allocation and its effects on function.

